# Prognostic value of fibrinogen change value in adrenocortical carcinoma patients

**DOI:** 10.1007/s12672-024-01197-5

**Published:** 2024-07-30

**Authors:** Chengquan Ma, Bin Yang, Quanzong Mao

**Affiliations:** 1 Department of Urology, Tianjin Medical University General Hospital, No 154. Anshan Road, Tianjin, 300052 China; 2 Department of Urology, The Affiliated Hospital of Qingdao University, Qingdao, China; 3 Department of Urology, Peking Union Medical College Hospital, Peking Union Medical College, Chinese Academy of Medical Sciences, Beijing, 100730 China

**Keywords:** Fibrinogen changes value, Adrenocortical carcinoma, Adrenal adenoma

## Abstract

**Purpose:**

The aim was to explore the preoperative and postoperative fibrinogen changes value (FCV) as a prognosis biomarker for in patients with adrenocortical carcinoma (ACC).

**Methods:**

We identified 42 patients with ACC and 190 patients with adrenal adenoma (AA) who underwent surgery at our institution between 2015 and 2023. Preoperative fibrinogen, postoperative fibrinogen and follow-up information of the patients were recorded and analysed. The relationship between FCV and overall survival (OS)/ relapse-free survival (RFS) was evaluated.

**Results:**

The mean level of preoperative and postoperative fibrinogen for ACC were 4.00 ± 1.64 g/L and 2.75 ± 0.59 g/L, respectively (p < 0.001). The mean level of preoperative and postoperative fibrinogen for AA were 2.79 ± 0.59 g/L and 2.71 ± 0.58 g/L, respectively (p = 0.144). In ACC, the lower FCV (≤ 1.25 g/L) showed a significantly poorer RFS than the higher (> 1.25 g/L) (p = 0.007); however, the lower FCV (≤ 1.25 g/L) showed no poorer OS than the higher (> 1.25 g/L) (p = 0.243). On multivariate survival analyses, FCV remained a predictor of RFS (HR 3.138).

**Conclusion:**

According to the data in this study, it can be said that FCV is correlated with prognosis of ACC. The FCV might be a new biomarker for predicting the RFS of ACC.

## Introduction

Adrenocortical carcinoma (ACC) is a rare malignancy with an incidence rate of about 0.7–2 per million per year [1]. Due to the insidious onset of ACC, the disease usually (over half of the patients) does not cause any clinical symptoms until it advances into progressive stages. Most patients with ACC have a poor prognosis, the expected 5-year survival rate of stage I patients is 80%, whereas that of stage IV patients is only 13% [2]. Even stage I to III tumors in patients with tumors after surgical treatment still have a high risk of recurrence, more than 50% of patients with ACC will develop recurrence within 5 years [3]. Mitotane can be used as an initial treatment for patients with ACC who have no surgical opportunity or cannot completely remove the tumor. Jason A Glenn conducted a retrospective review of 576 patients with adrenocortical carcinoma underwent resection of stage I–III adrenocortical carcinoma, found that 70% of patients developed disease recurrence [4].

There may be a variety of disorders in the body of cancer patients, especially inflammation, abnormal coagulation, abnormal nutrient consumption and so on. The relevant serological indicators indicate these disorders and reflect the body status of cancer patients. These include elevated plasma fibrinogen levels that reflect the inflammatory and clotting responses present in tumor patients that are associated with tumor progression. Fibrinogen is a key glycoprotein produced by the liver that functions as a non-specific acute phase reactant and is involved in inflammatory responses, clotting pathways, and tumor formation. Fibrinogen is involved in the proliferation and metastasis of malignant tumors by participating in the composition of extracellular matrix in the tumor microenvironment, being recognized by various integrins, activating the coagulation system and promoting the metastasis of circulating tumor cells [5].

Previous studies have used preoperative blood tests (neutrophil/lymphocyte ratio, platelet/lymphocyte ratio, red blood cell distribution width, mean platelet volume and plateletcrit) to predict the outcome of patients with ACC [6–8]. A large number of studies have raised the possibility of fibrinogen as a valuable marker for detecting cancer, informing prognoses, and monitoring treatment responses [9–11]. In a retrospective analysis of penile cancer, Ma et al. found that preoperative fibrinogen levels can be used as an independent prognostic marker to predict patient survival outcomes [12]. Masaaki Yamamoto et al. found that preoperative plasma fibrinogen level had the highest predictive value for recurrence among seven known hematological prognostic markers, and it would be useful for predicting prognosis after gastric cancer surgery [13]. However, the correlation between the postoperative fibrinogen changes and ACC is currently unknown. No studies have evaluated whether FCV predicts RFS and OS in postoperative ACC. In this study, we investigated the correlation between fibrinogen changes value (FCV) and ACC.

## Methods

We retrospectively evaluated the data of 42 patients with ACC and 190 patients with AA who underwent resection at Tianjin Medical University General Hospital and Peking Union Medical College Hospital between January 1, 2015 and January 1, 2023. Tumors were graded according to the Union for International Cancer Control TNM staging system. All pertinent laboratory, pathological results and medical data were obtained from hospital databases. Data obtained from patients’ routine preoperative or postoperative test results included plasma fibrinogen, age, sex, BMI, type of surgery, marginal condition, mitotane-based specific chemotherapy, R status and ki-67 index. Fibrinogen change value = preoperative fibrinogen level (within a week before surgery) minus postoperative fibrinogen level (1 month after surgery). Patients with coexisting hematologic diseases and those in the inflammatory phase were excluded. All patients with AA were nonfunctional adenomas. Within the preoperative fibrinogen and postoperative fibrinogen comparison, data obtained from the AA and ACC groups were evaluated using the t-test. The Kaplan–Meier product limit estimator was used to estimate the RFS and OS. A log-rank test was performed for comparison. For multivariate analysis, the Cox regression model was applied. The mean FCV was used to evaluate the prognostic RFS/OS relationship between fibrinogen change value and ACC.

Statistical analyses were performed using IBM SPSS Statistics for Windows, version 22.0 (IBM Corp, Armonk, NY). Statistical significance was set at p < 0.05.

## Results

Data from the 232 cases is presented in Table 1 (including the levels of fibrinogen, gender, age, BMI, and stage information). ACC consisted of 42 patients, with the diameter ranging from 3.3 cm to 19.2 cm and an average diameter of 6.42 ± 3.90 cm. While AA consisted of 190 patients, with the diameter ranging from 1.2 cm to 5.0 cm and an average diameter of 2.11 ± 0.60 cm.

**Table 1:** Patients’ characteristics

Groups	ACC	AA
Number of cases	42	190
Gender		
Male	22	91
Female	20	99
Age (mean, years)	62.93 ± 5.37	55.97 ± 11.36
BMI (mean, kg/m^2^)	24.68 ± 2.26	25.74 ± 4.99
Preoperative fibrinogen (mean, g/L)	4.00 ± 1.64	2.79 ± 0.59
Postoperative fibrinogen (mean, g/L)	2.75 ± 0.59	2.71 ± 0.58

*ACC* adrenocortical carcinoma, *AA* adrenal adenoma

The mean levels of preoperative and postoperative fibrinogen for ACC were 4.00 ± 1.64 g/L and 2.75 ± 0.59 g/L, respectively (p < 0.001). The mean FCV was 1.25 g/L. The mean level of preoperative and postoperative fibrinogen for AA were 2.79 ± 0.59 g/L and 2.71 ± 0.58 g/L, respectively (p = 0.144).

Patients with FCV ≤ 1.25 g/L had a higher tumor stage (p = 0.03), higher Ki-67 index (p = 0 0.005) compared to the patients with FCV > 1.25 g/L (Table 2).

**Table 2:** ACC patient characteristics with respect to FCV level

Variable	FCV > 1.25 (20 cases)	FCV ≤ 1.25 g/L (22 cases)	P value
Age (year)	62.13 ± 5.07	63.22 ± 5.42	0.43
Gender (female/male)	9/11	10/12	0.69
BMI	24.32 ± 2.23	24.77 ± 2.30	0.32
Tumor size (mm)	94 (35–192)	83 (33–140)	0.28
Tumor stage (n, %)			
I-II	17	12	**0.03**
III-IV	3	10	
Excess glucocorticoid secretion (n, %)	9 (45)	14 (64)	0.23
Ki-67 > 10% (%)	8 (40)	18 (82)	**0.005**

*FCV* fibrinogen changes value

At the time of last follow-up, 14 (35%) patients had developed recurrent disease after a median RFS of 63.41 months. For patients with FCV ≤ 1.25 g/L, the 5-year RFS was 25.0% and the 5-year OS was 33.3%. For patients with FCV > 1.25 g/L, the 5-year RFS was 90.9% and the 5-year OS was 61.6%.

**Fig. 1:**
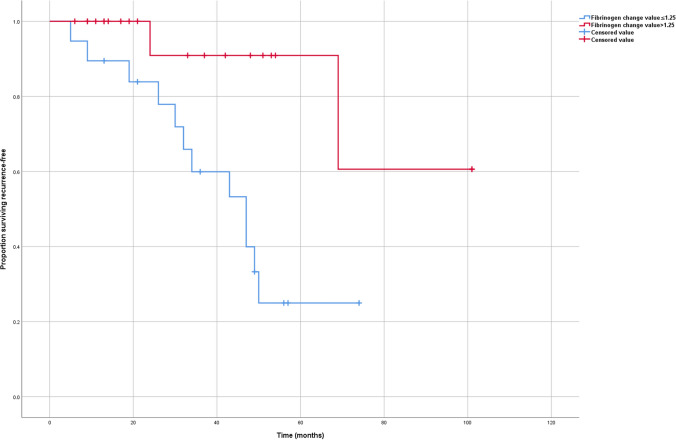
Relapse-free survival of adrenocortical carcinoma according to the FCV

**Fig. 2:**
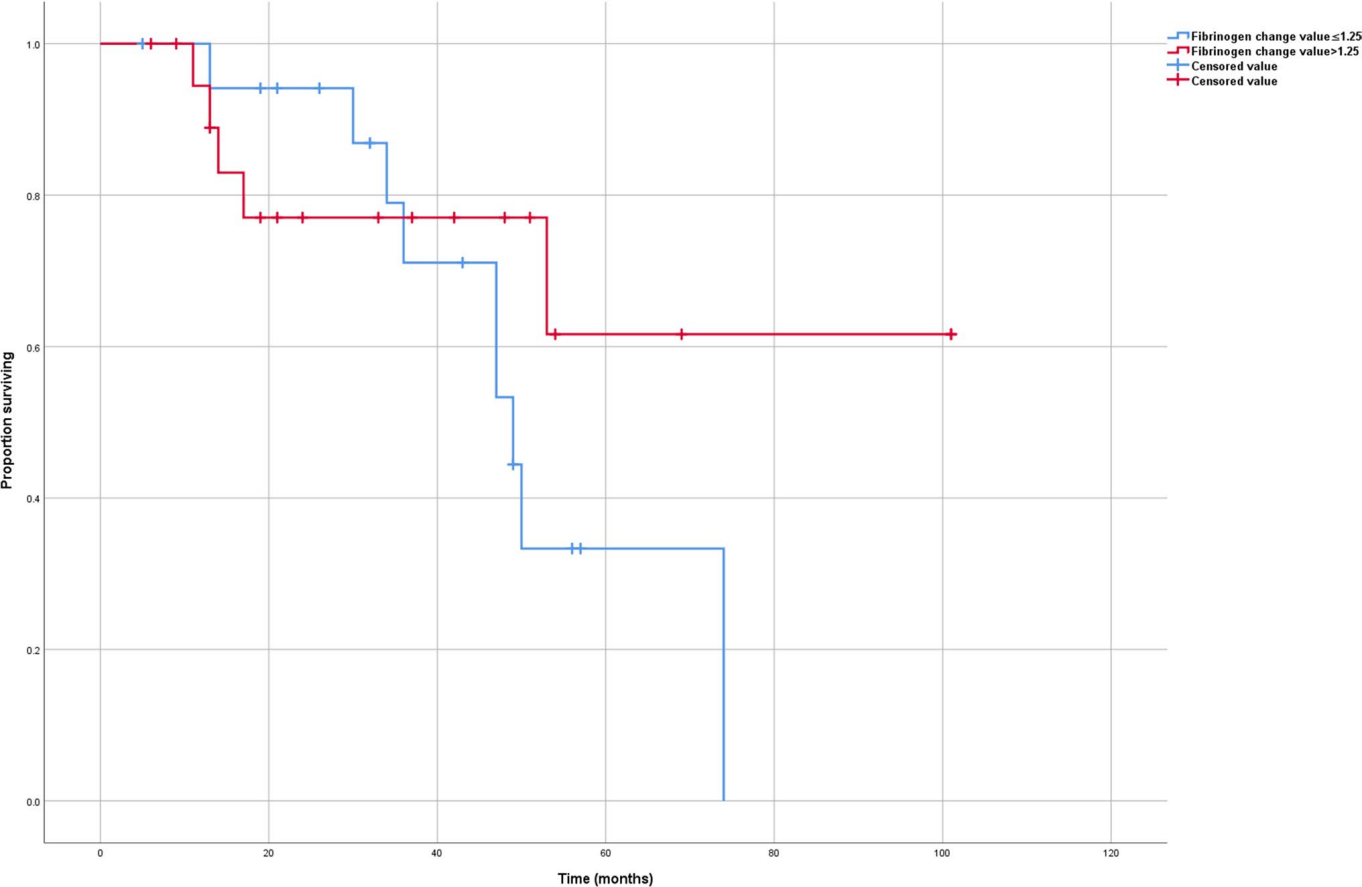
Overall survival of adrenocortical carcinoma according to the FCV

In ACC, the lower FCV (≤ 1.25 g/L) showed a significantly poorer RFS than the higher (> 1.25 g/L) (Fig. 1; p = 0.007); however, the lower FCV (≤ 1.25 g/L) showed no poorer OS than the higher (> 1.25 g/L) (Fig. 2; p = 0.243).

On multivariate survival analyses, tumor stage (HR 1.830, 95%CI 1.046 – 3.77), margin status (HR 1.501, 95%CI 1.210 – 4.063), Ki-67 > 10% (HR 2.125,95%CI 1.632 ~ 4.089) post-operative chemotherapy without mitotane (HR 1.620, 95%CI 1.133 – 7.125) as well as low FCV (HR 3.138, 95%CI 2.53 – 7.962) remained independent predictors of a shorter RFS (all P < 0.05; Table 3).

**Table 3:** Results of univariate and multivariable analysis for RFS

Parameters	Univariate analysis			Multivariable analysis		
	HR	95% CI	P-value	HR	95% CI	P-value
Gender (female vs male)	0.406	0.156 – 1.230	0.09			
Age (> 62.93vs ≤ 62.93)	0.995	0.726 – 2.619	0.987			
BMI (> 24.68vs ≤ 24.68)	1.042	0.993 – 1.024	0.693			
Excess glucocorticoid secretion (yes vs no)	**3.46**	**1.992 – 9.302**	**0.010**	1.442	0.946 – 8.720	0.061
Tumor stage (III-IV vs I-II)	**2.167**	**1.192 – 6.398**	**0.012**	**1.830**	**1.046 – 3.771**	**0.026**
Margin status (no-R0 vs R0)	**1.647**	**1.542 – 9.07**	**0.019**	**1.501**	**1.210 – 4.063**	**0.029**
Post-operative chemotherapy (no vs yes)	1.381	0.925 – 2.042	0.642			
Ki-67 > 10% (yes vs no)	**2.691**	**2.232 – 5.002**	**0.010**	**2.125**	**1.632 – 4.089**	**0.018**
Post-operative chemotherapy with mitotane (no vs yes)	**2.724**	**1.457 – 12.809**	**0.011**	**1.620**	**1.133 – 7.125**	**0.021**
FCV (≤ 1.25 vs F > 1.25)	**5.025**	**3.627 – 13.822**	**0.007**	**3.138**	**2.53 – 7.962**	**0.009**

Bold values indicate statistically significant

*FCV* fibrinogen changes value

## Discussion

Many previous studies have found correlations between preoperative fibrinogen levels and the clinicopathology and prognosis of tumors" for clearer expression. Generally, the higher the preoperative fibrinogen level, the more malignant the tumors and the worse the prognosis. Then, if fibrinogen is high before surgery, will the prognosis of significantly reduced after surgery be better? This article mainly answers this question. This study confirms these important findings. First, the mean postoperative fibrinogen was significantly lower than preoperative in ACC (2.75 ± 0.59 g/L vs. 4.00 ± 1.64 g/L, p < 0.001). The mean postoperative fibrinogen was not significantly lower than preoperative in AA (2.71 ± 0.58 g/L vs. 2.79 ± 0.59 g/L, p = 0.144). The cutoff value of FCV is estimated as 1.25 g/L. Elevated FCV is associated with better RFS. To our knowledge, this is the first study to consider the correlation between the FCV and ACC.

A major clinical feature of ACC is easy recurrence and poor prognosis, and the recurrence of ACC after surgery is mainly related to the late stage of the tumor and the surgical technique. If surgical resection of locally advanced ACC patients can be performed according to the principle of RO resection, if necessary, the adjacent organs that may be involved should be excised together, which may reduce the probability of local recurrence. Misperception of adrenal benign tumor before operation and failure to observe the principle of no tumor during operation will increase the risk of local recurrence after operation. The latest guidelines are the preferred open surgery for ACC surgery. For the failure to accurately determine ACC before surgery, minimally invasive surgical treatment, intraoperative tumor rupture, or failure to achieve R0 resection, is the important cause of local recurrence [14]. The reasons about ACC patients with a high risk for recurrence were explored, such as a ruptured capsule, large size or high-grade histology [15–17]. In patients without recurrence who tolerate mitotane therapy, it is recommended to administer the drug for at least 2 years [18] and up to 5 years [19]. It is difficult to determine whether the therapeutic benefits result from mitotane alone. In a meta-analysis including 6 retrospective studies, mitotane therapy resulted in improved mortality but not decreased recurrence [19].

In recent years, the relationship between hypercoagulability and malignant tumor progression has attracted extensive attention. Plasma fibrinogen is the main protein substance in the process of human coagulation. The coagulation function of malignant tumor patients is enhanced and the body is in a state of hypercoagulation, which makes it easy to form thrombosis. The fibrin principle plays an important role in inflammation and blood agglutination reactions. The formation and structural stability of fibrinogen are not only affected by individual genetics, environment, coagulation, thrombosis, pregnancy, inflammatory response, infection, diabetes, hypertension and other factors, but also have a certain relationship with the progression and prognosis of various malignant tumors.

Recent studies have shown that fibrinogen and pancreatic cancer [20], prostate cancer [21], laryngeal squamous cell carcinoma [22], renal cell carcinoma [23], endometrial cancer [24], penile cancer [24], small cell lung cancer [25], liver cancer [26], liposarcoma [27] and other tumors were related to the development or prognosis. This study found that preoperative plasma fibrinogen levels and postoperative fibrinogen changes have important value for the prognosis of ACC patients.

Fibrinogen is an essential landmark protein in the process of blood coagulation in the human body, and an acute-phase reactant in the inflammatory environment. It promotes the synthesis of pro-inflammatory cytokines, and the change of fibrinogen level can indicate systemic inflammation. It can also accumulate at the tumor site to form high expression and affect tumor progression [28]. Elevated fibrinogen plasma levels may be the result of hypercoagulability and hypoxia induced by tumor growth [29], may be the result of tumor cells themselves [30], or may be the result of inflammation mediating the cell as a host response to the tumor [31]. The possible mechanisms are as follows: (1) Fibrinogen can increase blood viscosity and peripheral resistance, promote red blood cell adhesion and thrombosis [32]; (2) Fibrinogen promotes tumor cell growth and angiogenesis through interaction with fibroblast growth factor-2 and vascular endothelial growth factor [32]. (3) Plasma fibrinogen is mainly produced in the liver through the activation of inflammatory cytokines interleukin-6 (IL-6) and IL-1β [33]. High plasma fibrinogen levels in patients may be related to excessive levels of inflammatory factors caused by tumors; (4) Fibrinogen may induce functional changes of stromal cells and inflammatory cells by integrating related cells, and promote cell proliferation and migration [34]. Previous studies have described the mechanism of hyperfibrinogenemia in patients with malignant tumors [35, 36]. Malignant cells typically have high levels of a fibrinogen receptor called intercellular adhesion molecule 1, and platelets also have a fibrinogen receptor called aIIb3 integrin. Malignant cells bind to platelets via fibrinogen. These aggregates form tumor clots that bind to the endothelium at the original site of the adrenal gland, adjacent tissues, and other organs and hide in the target organ to avoid immune system attack. Through this mechanism, fibrinogen plays a role in tumor progression, recurrence, and metastasis.

There are several restrictions on this study. First, because this study was retrospective in nature, it is unable to analyze attributable variables because of recollection bias. Second, it was known that prolonged exposure to high glucocorticoid levels affects fibrinogen quantities and function, potentially mimicking tumor-associated inflammatory reactions. Our results could have been affected to some extent by the tumor’s functional state and the resultant glucocorticoid overproduction. Third, since there are several variables that have a significant impact on the levels of systemic coagulation markers, even slight changes in these values might have an impact on prognostic evaluation. We anticipate that additional extensive and comprehensive patient investigations might more precisely demonstrate the relationship between FCV and the prognosis of ACC.

## Data Availability

The data could not be shared on open access; If needed during the review process, we could provide the datasets to the editor or editorial staff upon request (Chengquan Ma; machengquan@tmu.edu.cn).
